# Enhanced Barrier and Optical Properties of Inorganic Nano-Multilayers on PEN Substrate Through Hybrid Deposition

**DOI:** 10.3390/ma17236007

**Published:** 2024-12-08

**Authors:** Xiaojie Sun, Lanlan Chen, Wei Feng

**Affiliations:** 1 School of Materials Science and Engineering, Tianjin University, Tianjin 300350, China; 2 National Institute of Clean-and-Low-Carbon Energy, Beijing 102211, China; lanlan.chen@chnenergy.com.cn

**Keywords:** multilayer film, PEALD, ICP-CVD, Al_2_O_3_, WVTR

## Abstract

In this study, an inorganic multilayer barrier film was fabricated on the polyethylene naphthalate (PEN) substrate, which was composed of a SiO_2_ layer prepared by inductively coupled plasma chemical vapor deposition (ICP-CVD) and a Al_2_O_3_/ZnO nanolaminate produced by plasma-enhanced atomic layer deposition (PEALD). The multilayer composite film with a structure of 50 nm SiO_2_ + (4.5 nm Al_2_O_3_/6 nm ZnO) × 4 has excellent optical transmittance (88.1%) and extremely low water vapor permeability (3.3 × 10^−5^ g/m^2^/day, 38 °C, 90% RH), indicating the cooperation of the two advanced film growth methods. The results suggest that the defects of the SiO_2_ layer prepared by ICP-CVD were effectively repaired by the PEALD layer, which has excellent defect coverage. And Al_2_O_3_/ZnO nanolaminates have advantages over single-layer Al_2_O_3_ due to their complex diffusion pathways. The multilayer barrier film offers potential for encapsulating organic electronic devices that require a longer lifespan.

## 1. Introduction

Inorganic barrier thin films such as silicon oxide (SiO_2_), aluminum oxide (Al_2_O_3_), and zirconium oxide (ZrO_2_) on polymers have been widely used for the encapsulation of organic electronic devices to protect them from moisture penetration [1,2,3]. Given the lower temperature tolerance of polymer substrates, water vapor barriers should be manufactured at low temperatures [4,5]. The most common method for achieving high barrier properties appears to be ICP-CVD (inductively coupled plasma chemical vapor deposition). Films grow quickly but are prone to defects, particularly at low temperatures [6]. The PEALD (plasma-enhanced atomic layer deposition) method is considered a promising candidate for the growth of inorganic layers due to its excellent uniformity and conformity at low temperatures (<100 °C) [7,8,9]. However, the PEALD process is unproductive due to a rather low deposition rate.

Recent studies have been focused on multilayer structures, comprising alternating layers of different inorganic materials with individual layer thickness on a nanometer scale [10,11,12,13,14]. Choi fabricated the a-SiN_x_:H/SiO_x_N_y_/hybrid SiO_x_ hybrid barrier film on a polyethylene terephthalate (PET) substrate utilizing both PECVD and dip-coating processes, showing a high average transmittance of 88.8% in the spectral range of 400–700 nm and a water vapor transmission rate (WVTR) value of 7.3 × 10^−4^ g/m^2^/day [15]. Passlack studied the material properties of Al_2_O_3_-TiO_2_ nanolaminates deposited on PI-2611 films utilizing PEALD. Compared to alternative coating methods using the polymers parylene-C and benzocyclobutene, Al_2_O_3_-TiO_2_ nanolaminates with the correct thickness showed lower WVTR values and were therefore selected as encapsulation materials [16]. Currently, improved barrier performance has been reported by increasing the film thickness or the number of layers or optimizing the film formation process to improve the quality of the inorganic layer. However, the resulting multilayer barrier film may exhibit insufficient transparency and flexibility as the thickness increases [17].

In this study, a multilayer barrier film combining a SiO_2_ layer and a Al_2_O_3_/ZnO nanolaminate layer was fabricated. The SiO_2_ layer was prepared by ICP-CVD, which had been considered to contain defects or was not dense enough during low-temperature deposition. The Al_2_O_3_/ZnO nanolaminate layer was prepared by PEALD. It is well known that PEALD gives excellent conformal growth; thus, the PEALD layer can cover defects of the SiO_2_ layer. Meanwhile, the rapid growth of ICP-CVD makes it possible to overcome the shortcoming of the low growth efficiency of the PEALD method. The combination of the two advanced film growth methods helps to achieve high moisture barrier ability with WVTR value in the order of 10^−4^~10^–5^g/m^2^/day. Furthermore, compared with Al_2_O_3_ monolayer film, the Al_2_O_3_/ZnO nanolaminate was found to lower the WVTR by making a complicated diffusion path, resulting in an extremely low WVTR (3.3 × 10^−5^ g/m^2^/day) for the multilayer barrier film with a structure of 50 nm SiO_2_ + (4.5 nm Al_2_O_3_/6 nm ZnO) × 4.

## 2. Experimental Section

### 2.1. Preparation of the Multilayer Barrier Films

**Figure 1:**
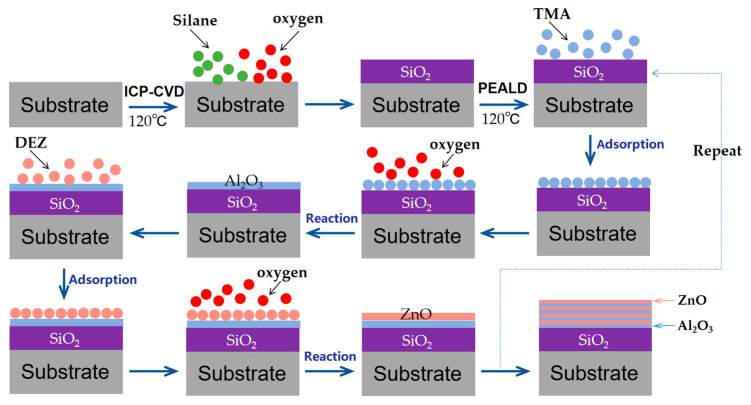
Schematic of the preparation of the multilayer barrier film.

The multilayer barrier film was prepared on a 100 μm thick PEN (DuPont Tejin films) substrate (Figure 1). The PEN substrate exhibits good thermal stability with the Tg of 122.7 °C. In order to ensure the thermal stability of the multilayer film, it is preferable for the deposition temperature to be below its glass transition temperature. The SiO_2_ thin film was prepared by ICP-CVD (Sentech SI 500, Sentech Instruments GmbH, Berlin, Germany) at 120 °C with a radio frequency power of 500 W. Silane and oxygen were used as reaction gases, and the thickness of the SiO_2_ film was set at 50 nm. Al_2_O_3_ and ZnO thin films were grown by PEALD (PICOSUN R200, Picosun Oy, Espoo, Finland) at 120 °C, and the intensity of the O_2_ plasma generator was 2700 W. For the preparation of Al_2_O_3_, trimethylaluminum (TMA) (purchased from Shanghai Dayuan New Materials Co., Ltd, Shanghai, China) and O_2_ plasma were used as precursors. Each ALD cycle consisted of 0.1 s exposure of TMA vapor, 6 s of N_2_ purge, 26 s exposure to O_2_ plasma, and then 6 s of Ar purge in sequence. The growth rate per cycle of Al_2_O_3_ was 0.124 nm/cycle. For the preparation of ZnO, diethylzinc (DEZ) (purchased from Shanghai Dayuan New Materials Co., Ltd, Shanghai, China) and O_2_ plasma were used as precursors. Each ALD cycle consisted of 0.1 s exposure of DEZ vapor, 6 s of N_2_-purge, 26 s exposure to O_2_ plasma, and then 6 s of Ar purge in sequence. The growth rate per cycle of ZnO was 0.1 nm/cycle. The Al_2_O_3_/ZnO nanolaminate layer contained 4 pairs of Al_2_O_3_ (6 nm, 4.5 nm, 3 nm) and ZnO (4.5 nm, 6 nm, 7.5 nm).

### 2.2. Characterization of the Barrier Films

The surface and cross-section microstructures of the barrier films were observed using an SEM (Nova NanoSEM 450, FEI Company, Hillsboro, OR, USA). The surface morphology and roughness of the barrier films were evaluated using atomic force microscopy (Dimension ICON, Bruker, Billerica, MA, USA). The cross-sectional morphology of the multilayer barrier film was observed using an HRTEM (JEM ARM200F, JEOL, Tokyo, Japan). Before TEM imaging, site-specific milling was performed by the focused ion beam (FIB) technique. The chemical bonding states of elements were investigated by XPS (Escalab 250Xi, Thermo Scientific, Waltham, MA, USA). The optical transmittance of the barrier films were measured using an ultraviolet-visible spectrophotometer (UV-3600, Shimadzu, Kyoto, Japan), and the measurement range was 400~1200 nm. The WVTR of the barrier films with a sample area of about 10 × 10 cm^2^ were obtained using a MOCON Aquatran 3 (Aquatran model 3, MOCON Inc., Brooklyn Park, MN, USA) at 38 °C and 90% relative humidity (RH).

## 3. Results and Discussion

**Figure 2:**
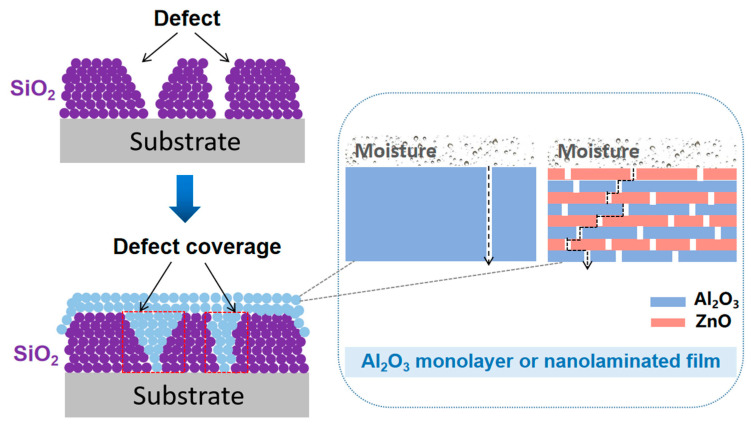
Schematic of the structure of multilayer barrier films and defect coverage in the SiO_2_ film.

In this work, a multilayer structure was formed by the sequential deposition of SiO_2_ (ICP-CVD) and a Al_2_O_3_ monolayer or nanolaminated film (PEALD). The nanolaminates consisted of four pairs of alternating Al_2_O_3_ and ZnO sublayers (Figure 2). The ICP-CVD process has the advantage of a fast growth rate, and thin films measuring tens or even hundreds of nanometers can be deposited in a short time. However, SiO_2_ films deposited by ICP-CVD at low temperature may have fine defects that allow moisture to pass through. The PEALD method has the properties of film-conforming growth. The filling of defects in the SiO_2_ film during the course of PEALD contributes to the formation of a perfect film with complete coverage. Therefore, the combination of ICP-CVD and PEALD can be an excellent candidate for producing high-quality barrier films. In addition, unlike traditional Al_2_O_3_ single films in the PEALD layer, the Al_2_O_3_/ZnO nanolaminate can achieve better moisture barrier ability due to the complicated diffusion path [18].

**Figure 3:**
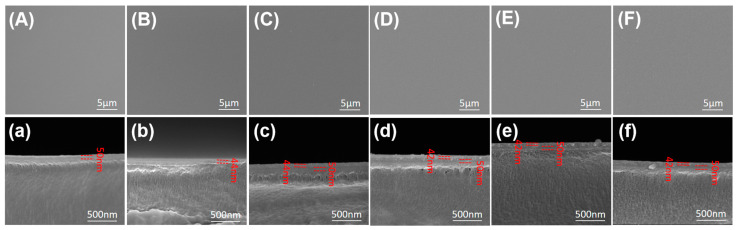
Surface and cross-sectional SEM images of SiO_2_ (**A**,**a**), Al_2_O_3_ (**B**,**b**), SiO_2_ + Al_2_O_3_ (**C**,**c**), SiO_2_ + (6 nm Al_2_O_3_/4.5 nm ZnO) × 4 (**D**,**d**), SiO_2_ + (4.5 nm Al_2_O_3_/6 nm ZnO) × 4 (**E**,**e**), and SiO_2_ + (3 nm Al_2_O_3_/7.5 nm ZnO) × 4 (**F**,**f**).

SEM images of the surface of different films are shown in Figure 3 (A, B, C, D, E, F). It is obvious that the surface of the films is very smooth and clean and the deposited films appear amorphous. From the cross-sectional SEM images (a, b, c, d, e, f) shown in Figure 3, the thickness of the SiO_2_ film is 50 nm, the thickness of the Al_2_O_3_ film with 350 deposition cycles is 44 nm, the total thickness of the SiO_2_ + Al_2_O_3_ film is 94 nm, and the total thickness of the SiO_2_ + Al_2_O_3_/ZnO films with different sublayer thicknesses is 92 nm. The boundary of the SiO_2_ layer and the PEALD layer can be clearly seen. However, the boundaries and respective thicknesses of the Al_2_O_3_ and ZnO sublayers are difficult to observe by SEM, which will be characterized by HRTEM in the next part.

**Figure 4:**
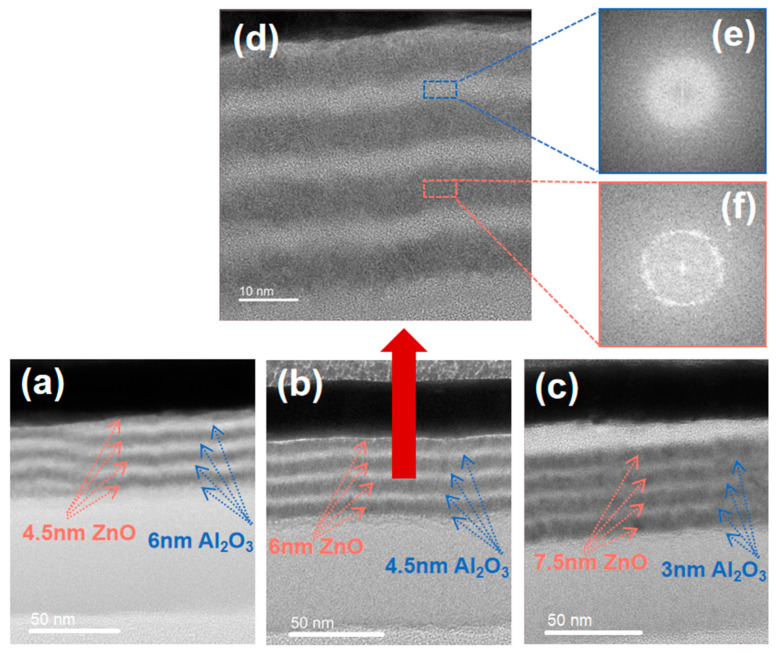
Low-resolution (**a**–**c**) and high-resolution (**d**) HRTEM cross-sectional images and FFT (**e**,**f**) of the SiO_2_ + Al_2_O_3_/ZnO multilayer films.

The cross-sectional structures of SiO_2_ + Al_2_O_3_/ZnO multilayer films with different sublayer thicknesses were observed by HRTEM to study the morphology of the films on a nanometer scale. Figure 4 shows the HRTEM images and fast Fourier transform (FFT) patterns. It can be seen that a clear Al_2_O_3_/ZnO multilayer stacking structure is obtained on the SiO_2_ surface. The FFT patterns clearly show that the Al_2_O_3_ underlayer is amorphous and the ZnO underlayer is crystalline. Similar results have been reported in previous studies [19,20,21]. The introduction of ZnO underlayers is expected to be beneficial to achieve better permeation pathways compared to pure Al_2_O_3_ film. At the same time, the further crystallization of ZnO should be suppressed by the Al_2_O_3_ deposition. This means that the water vapor permeation caused by the nanocrystal can be hindered through grain boundaries. Another notable observation in Figure 4d is that the interface between the Al_2_O_3_ and ZnO sublayers appears very smooth. Previous reports on nanolaminates showed that a mixed phase can form at the sublayer boundaries, which was shown to be denser than separate layers, and densification at the interfaces was beneficial in preventing moisture penetration [22,23].The mixed phase formed at the sublayer boundaries in our Al_2_O_3_/ZnO nanolaminate was further confirmed by the characterization of XPS.

**Figure 5:**
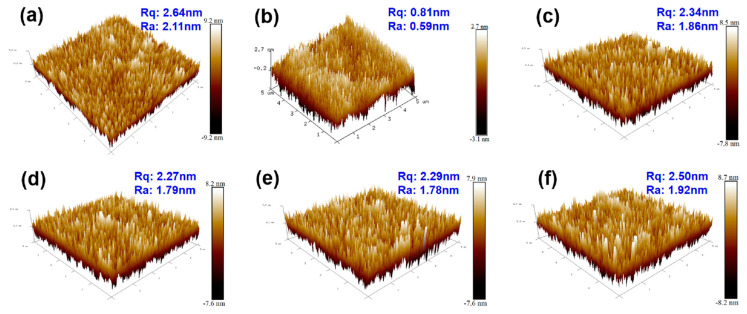
Images and surface roughness measurements via AFM. (**a**) SiO_2_, (**b**) Al_2_O_3_, (**c**) SiO_2_ + Al_2_O_3_, SiO_2_ + (6 nm Al_2_O_3_/4.5 nm ZnO) × 4 (**d**), SiO_2_ + (4.5 nm Al_2_O_3_/6 nm ZnO) × 4 (**e**), and SiO_2_ + (3 nm Al_2_O_3_/7.5 nm ZnO) × 4 (**f**).

AFM is used to examine the surface properties of thin films deposited on PEN substrate. The root mean square roughness (*Rq*) and average roughness (*Ra*) are roughness parameters that indicate the surface topography. A lower *Rq* and *Ra* generally indicate a smoother surface. In this work, *Rq* and *Ra* values were obtained from the scanned images over 5 × 5 μm^2^. As shown in Figure 5a, the *Rq* and *Ra* of the SiO_2_ layer are 2.64 nm and 2.11 nm, respectively. In comparison, the Al_2_O_3_ layer exhibits a very smooth surface with an *Rq* of 0.81 nm and an *Ra* of only 0.59 nm (Figure 5b). It has been reported that the surface of thin films deposited by PEALD at low temperature is smooth [24,25]. For the SiO_2_ + Al_2_O_3_ (Figure 5c) and SiO_2_ + Al_2_O_3_/ZnO multilayer films (Figure 5d–f), they both have a lower *Rq* and *Ra* value than the SiO_2_ layer due to the defects of the SiO_2_ layer being effectively covered by the PEALD layer. Thus, the combination of SiO_2_ (ICP-CVD) and the Al_2_O_3_ monolayer or nanolaminate (PEALD) will help to obtained improved surface smoothness. This can physically impede the movement of water molecules and limit their access to the underlying PEN substrate, which is more favorable for their use as effective moisture barriers. It agrees with the subsequent WVTR data.

**Figure 6:**
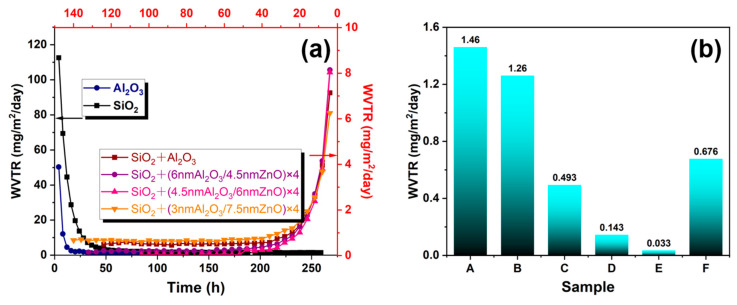
(**a**) Test curves of water vapor permeability for different films measured by a MOCON Aquatran 3; (**b**) WVTR values of (A) SiO_2_, (B) Al_2_O_3_, (C) SiO_2_ + Al_2_O_3_, and (D) SiO_2_ + Al_2_O_3_/ZnO multilayer films.

Figure 6 presents the results of the WVTR measurement for the permeation barrier layers prepared for the experiments. In the case of single layers, the Al_2_O_3_ layer shows a better permeation barrier property than the SiO_2_ layer. The WVTR of the 44 nm Al_2_O_3_ layer is 1.26 × 10^−3^ g/m^2^/day, and that of the 50 nm SiO_2_ layer is 1.46 × 10^−3^ g/m^2^/day. This result suggests that a thin SiO_2_ layer deposited by ICP-CVD at low temperature would possess defects as a result of passes of water vapor penetration. The barrier ability of Al_2_O_3_ with the thickness of 44 nm deposited by PEALD is not excellent by itself. The SiO_2_(50 nm)/Al_2_O_3_(44 nm) film shows a lower WVTR value than single films with the order of 10^−4^ g/m^2^/day. The improved barrier property of SiO_2_ + Al_2_O_3_ film is attributed to the formation of a perfect film with full coverage by the combination of ICP-CVD and PEALD. In order to further improve the barrier performance, SiO_2_(50 nm)/(Al_2_O_3_/ZnO)(42 nm) multilayer structures with different sublayer thicknesses are investigated. It can be seen that the WVTR value of multilayer films decreased as the thickness of the ZnO sublayer increased from 4.5 to 6 nm. However, when the thickness of the ZnO sublayer further increased to 7.5 nm, the WVTR value of the multilayer film increases, which is because of the relatively poor water vapor barrier ability of ZnO with a crystalline nature. As a result, the SiO_2_ + (4.5 nm Al_2_O_3_/6 nm ZnO) × 4 multilayer film exhibits the lowest WVTR value of 3.3 × 10^−5^ g/m^2^/day.

The reason why the SiO_2_ + Al_2_O_3_/ZnO multilayer structure shows better moisture barrier property than the single-layer Al_2_O_3_ film may be explained by the mismatch of permeation paths or channels among the different layers. Generally, in the single-layer Al_2_O_3_ film, paths or channels are formed via defect connection, which may allow for moisture to continually penetrate to the inside. Unlike the single-layer film, defects in the SiO_2_ + Al_2_O_3_/ZnO multilayer structure cannot be directly connected to form full paths or channels. The defects located in each layer become mismatched because each layer has different structures due to different oxide compositions. Therefore, it is difficult to form a complete connection from the inner layer to the external atmosphere throughout the depth of the film, leading to the lowest WVTR value in the SiO_2_ + Al_2_O_3_/ZnO multilayer film [26,27].

**Figure 7:**
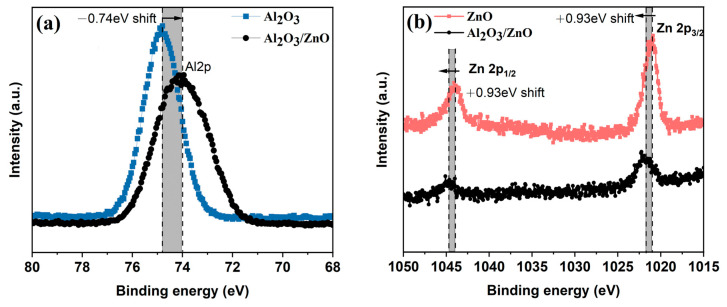
XPS analysis of Al_2_O_3_ and Al_2_O_3_/ZnO nanolaminate (**a**), ZnO and Al_2_O_3_/ZnO nanolaminate (**b**).

Based on the above analysis, the thickness of each sublayer of Al_2_O_3_ and ZnO in the Al_2_O_3_/ZnO nanolaminate is set at 4.5 nm and 6 nm, respectively. Figure 6 shows the XPS results for single Al_2_O_3_, ZnO and Al_2_O_3_/ZnO nanolaminate. Compared to the Al_2_O_3_, the lower Al 2p binding energy shift (−0.74 eV) occurs in the Al_2_O_3_/ZnO nanolaminate (Figure 7a). In the case of Zn 2p peaks, a 0.93 eV binding energy shift is observed in the Al_2_O_3_/ZnO nanolaminate (Figure 7b). Similar core-level shifts in a Al_2_O_3_/ZnO nanolaminate have also been reported in other studies [28,29]. These results suggest that the shifts in the core level come from chemical bonding at the Al_2_O_3_/ZnO interfaces. This chemical bonding or aluminate phase at interfaces between Al_2_O_3_ and ZnO sublayers contributes to form a denser structure, and the denser film structure reduces the number and size of pores and channels through which moisture can penetrate. In addition, this chemical bonding or aluminate phase at interfaces between Al_2_O_3/_ZnO sublayers contributes to the formation of a film with a strong binding force at the interface. As a result, the penetration of moisture, which progresses along the Al_2_O_3_/ZnO interface, may be inhibited [23]. The above analysis results are consistent with those of HRTEM and WVTR data.

**Figure 8:**
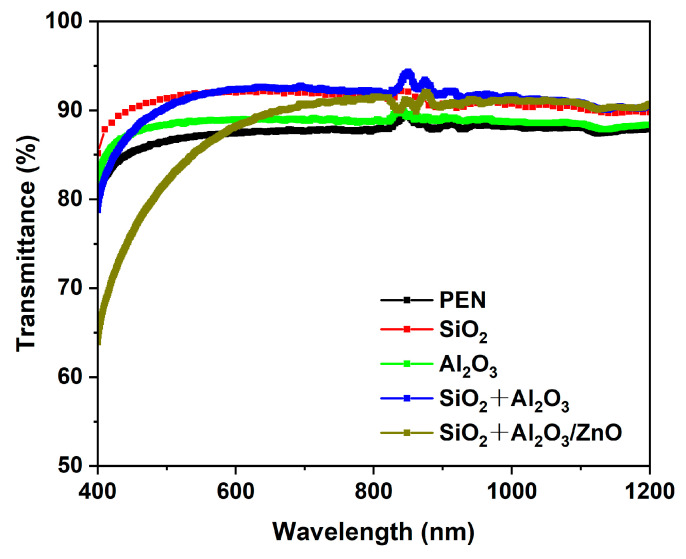
Optical transmittance of the films by UV-vis measurements.

The optical properties of the barrier films are also important for use on transparent, flexible organic electronic devices. The optical transmittance in the range of 400–1200 nm of pristine PEN, a single SiO_2_, and Al_2_O_3_, SiO_2_ + Al_2_O_3_, and SiO_2_ + Al_2_O_3_/ZnO multilayer films is shown in Figure 8. Higher average transparency over the entire wavelength range was observed for different barrier structures compared to the pristine PEN substrate (Tr 87.5%). The individual SiO_2_ and Al_2_O_3_ films showed an average transmittance value of 90.9% and 88.5% between 400 and 1200 nm wavelengths, respectively. The SiO_2_ + Al_2_O_3_ film showed the highest average transmittance (91.1%) among the four barrier structures, whereas the SiO_2_ + Al_2_O_3_/ZnO multilayer film showed lower transmittance (88.1%). Although the introduction of the Al_2_O_3_/ZnO nanolaminate reduced the transparency of the multilayer film, it still improved over the original PEN substrate. It is worth noting that the Al_2_O_3_/ZnO nanolaminate can significantly improve the barrier properties against moisture. However, the addition of the Al_2_O_3_/ZnO nanolaminate onto the SiO_2_ layer caused a lower transmittance than the SiO_2_ + Al_2_O_3_ film. On one hand, the addition of more layers can lead to an increase in light scattering at the interfaces. On the other hand, the Al_2_O_3_/ZnO layer has a lower transmittance than Al_2_O_3_ due to the grain boundaries in crystalline ZnO causing a scattering of light. This is a trade-off, as flexible organic electronic devices require both good barrier properties and optical transmittance. So, the nanolaminate sublayers should be optimized in terms of thickness to maintain the desired barrier performance without sacrificing the optical transmittance.

## 4. Conclusions

In summary, a high-performance multilayer SiO_2_ + Al_2_O_3_/ZnO barrier film was fabricated on a PEN substrate by combining ICP-CVD and PEALD. The multilayer barrier film with 50 nm SiO_2_ and (4.5 nm Al_2_O_3_/6 nm ZnO) × 4 nanolaminate layers achieved high moisture barrier ability with WVTR values in the order of 10^−5^ g/m^2^/day. The excellent barrier performance of the multilayer film is due to the defect coverage and complicated diffusion path. In addition, the average light transmittance of the multilayer barrier film is 88.1% between the 400 and 1200 nm wavelength, which ensures its optical superiority. This work provides a practical strategy for flexible encapsulation applications of large-area electronic devices.

Notably, the WVTR value of the optimized SiO_2_ + Al_2_O_3_/ZnO film is comparable to or better than the previously reported lowest WVTR values of inorganic films based on single-layer or nanolaminate structures [30,31,32,33,34]. The main advantages of our work are as follows: (1) First, there is a large area of 10 × 10 cm^2^. The optimal preparation technology of inorganic/inorganic multilayer composite film in this paper can ensure the uniformity and density of the film over a larger dimension, while most of the barrier films reported in the literature are of a relatively small size [30,31,32,33,34]. (2) Second, there is low WVTR. In our work, the WVTR of the multilayer barrier film with a sample area of about 10 × 10 cm^2^ was obtained as 3.3 × 10^−5^ g/m^2^/day using a MOCON Aquatran 3 at 38 °C and 90% relative humidity (RH). However, most of the barrier films reported in the literature are limited by their small size and are usually tested using the calcium corrosion method, and the test results may be influenced by human factors. In addition, some of the lower WVTR results were obtained under low temperature or/and low relative humidity conditions (such as [30,32]). (3) Third, there is low film thickness (<100 nm). In this study, the multilayer barrier film obtained good water vapor barrier property with a thickness below 100 nm. However, in some of the literature reports, it has been reported that the thickness required for the film to obtain a comparable water vapor barrier property is very thick (such as [30,32]). This will lead to a cumbersome preparation process and a significant increase in cost.

## Data Availability

The original contributions presented in this study are included in the article. Further inquiries can be directed to the corresponding authors.

## References

[1] Lee Y., Seo S., Oh I.-K., Lee S., Kim H. (2019). Effects of O_2_ plasma treatment on moisture barrier properties of SiO_2_ grown by plasma-enhanced atomic layer deposition. Ceram. Int..

[2] Hoffmann L., Theirich D., Pack S., Kocak F., Schlamm D., Hasselmann T., Fahl H., Räupke A., Gargouri H., Riedl T. (2017). Gas diffusion barriers prepared by spatial atmospheric pressure plasma enhanced ALD. ACS Appl. Mater..

[3] Duan Y., Sun F., Yang Y., Chen P., Yang D., Duan Y., Wang X. (2014). Thin-film barrier performance of zirconium oxide using the lowtemperature atomic layer deposition method. ACS Appl. Mater..

[4] Nehm F., Klumbies H., Richter C., Singh A., Schroeder U., Mikolajick T., Mönch T., Hoßbach C., Albert M., Bartha J.W. (2015). Breakdown and protection of ALD moisture barrier thin films. ACS Appl. Mater..

[5] Park S., Jeonga Y., Baeka Y., Kim L., Jang J., Kim Y., An T., Nam S., Kim H., Jang J. (2017). Reduced water vapor transmission rates of low-temperature solution-processed metal oxide barrier films via ultraviolet annealing. Appl. Surf. Sci..

[6] Revuri P.K., Tripathi D.K., Martyniuk M., Silva K.K.M.B.D., Putrino G., Keating A., Faraone L. Silicon and silicon dioxide thin films deposited by ICPCVD at low temperature and high rate for MEMS applications. Proceedings of the 2018 Conference on Optoelectronic and Microelectronic Materials and Devices (COMMAD).

[7] Preischel F., Zanders D., Berning T., Kostka A., Rogalla D., Bock C., Devi A. (2023). Interplay of precursor and plasma for the deposition of HfO_2_ via PEALD: Film growth and dielectric properties. Adv. Mater..

[8] Lee S., Han G., Kim K.H., Shim D., Go D., An J. (2024). High-performance TiO_2_/ZrO_2_/TiO_2_ Thin film capacitor by plasma assisted atomic layer annealing. ACS Appl. Mater..

[9] Kim B., Lee N., Lee J., Park T., Park H., Kim Y., Jin C., Lee D., Kim H., Jeon H. (2021). Remote plasma enhanced atomic layer deposition of titanium nitride film using metal organic precursor (C_12_H_23_N_3_Ti) and N_2_ plasma. Appl. Surf. Sci..

[10] Tseng M.H., Yu H.H., Chou K.Y., Jou J.H., Lin K.L., Wang C.C., Tsai F.-Y. (2016). Low-temperature gas-barrier films by atomic layer deposition for encapsulating organic light-emitting diodes. Nanotechnology.

[11] Shin S.U., Ryu S.O. (2021). Optical Transmittance Improvements of Al_2_O_3_/TiO_2_ multilayer OLED encapsulation films processed by atomic layer deposition. J. Electron..

[12] Li M., Xu M., Zou J., Tao H., Wang L., Zhou Z., Penng Z. (2016). Realization of Al_2_O_3_/MgO laminated structure at low temperature for thin film encapsulation in organic light-emitting diodes. Nanotechnology.

[13] Satoh R., Ro T., Heo C.J., Lee G.H., Xianyu W., Park Y., Park J., Lim S.-J., Leem D.-S., Bulliard X. (2017). Bi-layered metal-oxide thin films processed at low-temperature for the encapsulation of highly stable organic photo-diode. Org. Electron..

[14] Doyle S., Ryan L., McCarthy M.M., Modreanu M., Schmidt M., Laffir F., Povey I.M., Pemble M.E. (2022). Combinatorial ALD for the growth of ZnO/TiO_2_ nanolaminates and mixed ZnO/TiO_2_ nanostructured films. Mater. Adv..

[15] Lim K.Y., Kim H.H., Noh J.H., Tak S.H., Yu J.W., Choi W.K. (2022). Thin film encapsulation for quantum dot lightemitting diodes using a-SiNx:H/SiOxNy/hybrid SiOx barriers. RSC Adv..

[16] Passlack U., Simon N., Bucher V., Harendt C., Stieglitz T., Burghartz J.N. (2023). Flexible ultrathin chip-film patch for electronic component integration and encapsulation using atomic layer-deposited Al_2_O_3_-TiO_2_ nanolaminates. ACS Appl. Mater..

[17] Bulusu A., Singh A., Wang C.Y., Dindar A., Fuentes-Hernandez C., Kim H., Cullen D., Kippelen B., Graham S. (2015). Engineering the mechanical properties of ultrabarrier films grown by atomic layer deposition for the encapsulation of printed electronics. J. Appl. Phys..

[18] Jeong E.G., Han Y.C., Im H.G., Bae B.S., Choi K.C. (2016). Highly reliable hybrid nano-stratified moisture barrier for encapsulating flexible OLEDs. Org. Electron..

[19] Osorio D., Lopez J., Tiznado H., Farias M.H., Hernandez-Landaverde M.A., Ramirez-Cardona M., Yañez-Limon J.M., Gutierrez J.O., Caicedo J.C., Zambrano G. (2020). Structure and surface morphology effect on the cytotoxicity of [Al_2_O_3_/ZnO]n/316L SS nanolaminates growth by atomic layer deposition (ALD). Crystals.

[20] Choi D., Kim S.J., Lee J.H., Chung K.B., Park J.S. (2012). A study of thin film encapsulation on polymer substrate using low temperature hybrid ZnO/Al_2_O_3_ layers atomic layer deposition. Curr. Appl. Phys..

[21] Lin Y.Y., Chang Y.N., Tseng M.H., Wang C.C., Tsai F.Y. (2015). Air-stable flexible organic light-emitting diodes enabled by atomic layer deposition. Nanotechnology.

[22] Oh J., Shin S., Park J., Ham G., Jeon H. (2016). Characteristics of Al_2_O_3_/ZrO_2_ laminated films deposited by ozone-based atomic layer deposition for organic device encapsulation. Thin Solid Film..

[23] Meyer J., Schmidt H., Kowalsky W., Riedl T., Kahn A. (2010). The origin of low water vapor transmission rates through Al_2_O_3_/ZrO_2_ nanolaminate gas-diffusion barriers grown by atomic layer deposition. Appl. Phys. Lett..

[24] Castillo-Saenz J., Nedev N., Valdez-Salas B., Curiel-Alvarez M., Mendivil-Palma M.I., Hernandez-Como N., Martinez-Puente M., Mateos D., Perez-Landeros O., Martinez-Guerra E. (2021). Properties of Al_2_O_3_ thin films grown by PE-ALD at low temperature using H_2_O and O_2_ plasma oxidants. Coatings.

[25] Gebhard M., Mai L., Banko L., Mitschker F., Hoppe C., Jaritz M., Kirchheim D., Zekorn C., de los Arcos T., Grochla D. (2018). PEALD of SiO_2_ and Al_2_O_3_ thin films on polypropylene: Investigations of the film growth at the interface, stress, and gas barrier properties of dyads. ACS Appl. Mater..

[26] Kang K.S., Jeong S.Y., Jeong E.G., Choi K.C. (2020). Reliable high temperature, high humidity flexible thin film encapsulation using Al_2_O_3_/MgO nanolaminates for flexible OLEDs. Nano Res..

[27] Jeong E.G., Kwon S., Han J.H., Im H.-G., Bae B.-S., Choi K.C. (2017). Mechanically enhanced hybrid nano-stratified barrier with defect suppression mechanism for highly reliable flexible OLEDs. Nanoscale.

[28] Cheun H., Fuentes-Hernandez C., Shim J., Fang Y., Cai Y., Li H., Sigdel A.K., Meyer J., Maibach J., Dindar A. (2012). Oriented growth of Al_2_O_3_:ZnO nanolaminates for use as electron-selective electrodes in inverted polymer solar cells. Adv. Func..

[29] Kwon J.H., Jeon Y., Choi S., Park J.W., Kim H., Choi K.C. (2017). Functional design of highly robust and flexible thin-film encapsulation composed of quasi-perfect sublayers for transparent, flexible displays. ACS Appl. Mater..

[30] Weng Y.L., Chen G.X., Zhou X.T., Zhang Y.A., Yan Q., Guo T.L. (2023). Design and fabrication of PDMS/Al_2_O_3_ hybrid flexible thin films for OLED encapsulation applications. ACS Appl. Polym. Mater..

[31] Eom J.H., Cho T.Y., Cho S.K. (2024). Performance of multifunctional antibacterial moisture barrier films with different Zn/Al ratios fabricated by plasma enhanced atomic layer deposition. Appl. Surf. Sci..

[32] Lee S., Jeon Y., Oh S.J., Lee S.-W., Choi K.C., Kim T.-S., Kwon J.H. (2023). Study of mechanical degradation of freestanding ALD Al_2_O_3_ by a hygrothermal environment and a facile protective method for environmentally stable Al_2_O_3_: Toward highly reliable wearable OLEDs. Mater. Horiz..

[33] Han J.-H., Kim D.-Y., Lee S., Yang H.L., Park B.H., Park J.-S. (2021). A study on the growth mechanism and gas diffusion barrier property of homogeneously mixed silicon–tin oxide by atomic layer deposition. Ceram. Int..

[34] Kim L.H., Kim K., Park S., Jeon Y.J., Kim H., Chung D.S., Kim S.H., Park C.E. (2014). Al_2_O_3_/TiO_2_ nanolaminate thin film encapsulation for organic thin film transistors via plasma-enhanced atomic layer deposition. ACS Appl. Mater. Interfaces.

